# Bridging the HIV treatment gap in Nigeria: examining community antiretroviral treatment models

**DOI:** 10.1002/jia2.25108

**Published:** 2018-04-20

**Authors:** Edward A Oladele, Okikiolu A Badejo, Christopher Obanubi, Emeka F Okechukwu, Ezekiel James, Golden Owhonda, Onuche I Omeh, Moyosola Abass, Olubunmi R Negedu‐Momoh, Norma Ojehomon, Dorothy Oqua, Satish Raj‐Pandey, Hadiza Khamofu, Kwasi Torpey

**Affiliations:** ^1^ Prevention, Care and Treatment Department FHI 360 (Family Health International) Abuja Nigeria; ^2^ Institute of Tropical Medicine (ITM) Antwerp Belgium; ^3^ Office of HIV/AIDS and TB U.S. Agency for International Development (USAID) Abuja Nigeria; ^4^ Ministry of Health Rivers State Nigeria; ^5^ Howard University Global Initiative Abuja Nigeria; ^6^ Monitoring and Evaluation Department FHI 360 (Family Health International) Abuja Nigeria; ^7^ Department of Laboratory Services FHI 360 (Family Health International) Abuja Nigeria; ^8^ Strategic Information USAID Abuja Nigeria; ^9^ FHI 360 (Family Health International) Abuja Nigeria; ^10^ College of Health Sciences University of Ghana Accra Ghana

**Keywords:** community‐based, antiretroviral therapy, Universal Access, community‐models, local government areas, HIV positive

## Abstract

**Introduction:**

Significant gaps persist in providing HIV treatment to all who are in need. Restricting care delivery to healthcare facilities will continue to perpetuate this gap in limited resource settings. We assessed a large‐scale community‐based programme for effectiveness in identifying people living with HIV and linking them to antiretroviral treatment.

**Methods:**

A retrospective secular trend study of 14 high burden local government areas of Nigeria was conducted in which two models of community antiretroviral treatment delivery were implemented: Model A (on‐site initiation) and Model B (immediate referral) clusters. Model A cluster offered services within communities, from HIV diagnosis to immediate antiretroviral therapy initiation and some follow‐up. Model B cluster offered services for HIV diagnosis up to baseline evaluation and provided referral for antiretroviral therapy initiation to nearest health facility providing HIV services. For controls, we selected and cluster‐matched 34 local government areas where community antiretroviral treatment delivery was not implemented. Outcomes of interest were: the number of people identified as HIV positive and the number of HIV‐positive individuals started on antiretroviral treatment; from June 2014 to May 2016. We used interrupted time‐series analysis to estimate outcome levels and trends across the pre‐and post‐intervention periods.

**Results:**

Before community antiretrovial treatment introduction, Model A cluster identified, per 100,000 catchment population, 500 HIV‐positives (95% CI: 399.66 to 601.41) and initiated 216 HIV‐positives on antiretroviral treatment (95% CI: 152.72 to 280.10). Model B cluster identified 32 HIV‐positives (95% CI: 25.00 to 40.51) and initiated 8 HIV‐positives on antiretroviral treatment (95% CI: 5.54 to 10.33). After commART introduction, Model A cluster showed an immediate significant increase in 744 HIV‐positive persons (*p* = 0.00, 95% CI: 360.35 to 1127.77) and 560 HIV‐positives initiated on treatment (*p* = 0.00, 95% CI: 260.56 to 859.64). Model B cluster showed an immediate significant increase in 30 HIV‐positive persons identified (*p* = 0.01, 95% CI: 8.38 to 51.93) but not in the number of HIV‐positives initiated on treatment. Model B cluster showed increased month‐on‐month trends of both outcomes of interest (3.4, *p* = 0.02, 95% CI: 0.44 to 6.38).

**Conclusion:**

Both community‐models had similar population‐level effectiveness for rapidly identifying people living with HIV but differed in effectively transitioning them to treatment. Comprehensiveness, integration and attention to barriers to care are important in the design of community antiretroviral treatment delivery.

## 1 Introduction

Globally, 36.7 million people were living with HIV by the end of 2015, and sub‐Saharan Africa (SSA) accounted for 70% of this burden 1. Despite the disproportionate burden, less than 60% of the people living with HIV in SSA have access to antiretroviral therapy (ART) 1. Access to ART for everyone who requires it is central to the attainment of the global goal of eliminating HIV. The conventional model in SSA of providing access to ART through healthcare facilities has been inadequate to achieve required treatment coverage 2, 3. As the world strives to achieve the ambitious treatment targets set forth by the joint United Nations Programme on HIV/AIDS (UNAIDS) 4 to eliminate the disease, alternate HIV‐care delivery models are becoming more relevant 5. One such model is community‐ART delivery.

Community‐ART (commART) refers to various modalities of decentralizing HIV‐related care and treatment outside of health facilities. The modalities vary based on adaptations to local contexts and needs 6. The main thrust of commART is on engaging and capacitating community members to deliver HIV‐related treatment services directly within communities through mobile clinics, primary care centres, homes and sexual networks (a group of people who are connected to one another by having sexual relationships) 7. Despite variations in implementation approaches, several studies have examined the effectiveness of commART interventions 7, 8, 9, 10, 11, 12, 13, 14. Findings from these studies continue to highlight the importance of commART to global goals as well as the need to evolve more context‐specific approaches for bringing ART closer to the people. In this vein, recent World Health Organization (WHO) HIV treatment guidelines 15 have contained strong recommendations on implementing locally driven approaches to commART.

Nigeria, together with South Africa, accounts for over 40% of HIV burden in SSA 16, 17.

However, less than 25% of all estimated people living with HIV in Nigeria were accessing antiretroviral treatment at the end of 2014 18. After the beginning of Nigeria's ART programme in 2002, ART scale‐up has occurred mainly through decentralization from tertiary facilities to secondary and primary levels of facility‐based delivery. Health facilities providing ART services increased from 25 tertiary facilities in 2002 to over 1000 facilities in 2013 consisting of primary, secondary and tertiary facilities 19. These facilities were mainly public and large private not‐ for‐profit ones. Since 2013, however, the number of private‐for‐profit health facilities providing HIV services as part of the national programme has significantly increased. Substantial barriers to accessing regular healthcare from health facilities in Nigeria, however, limit the national goal to provide HIV treatment access to at least 80% of those who need it 20. Progress may continue to be slow without adaptations to this current strategy of ART scale‐up.

Given that marked variations exist in HIV prevalence and treatment coverage across Nigerian subnational units 21, 22, it is imperative that interventions become context‐specific at these levels. We report outcomes of two variants of an extensive commART programme implemented in 14 local government areas (LGAs) across four of Nigeria's 36 states. Our primary hypothesis was that this commART programme would increase the level and trend of identification of people living with HIV as well as treatment access and uptake within implementing LGAs.

## 2 Methods

### 2.1 Study design and setting

We conducted a secular trend study, using interrupted time‐series analysis, to evaluate the population‐level effect of commART on the identification of people living with HIV and uptake of ART services across Nigerian LGAs.

Nigeria is administratively divided into 36 states and each state further divided into LGAs. The states and LGAs may be the equivalent of province and districts in some other countries. HIV care and treatment in these LGAs is freely provided through a mix of public and private healthcare facilities by the Government of Nigeria (GON). The U.S. President's Emergency Plan for AIDS Relief (PEPFAR) has been providing financial and technical support to GON's effort through its various PEPFAR implementing agencies.

In 2015, PEPFAR prioritized 32 highest‐burden LGAs across Nigeria for intensive scale‐up of HIV services, and the United States Agency for International Development (USAID), a PEPFAR implementing agency, provided funding to support 14. Beginning in June 2015 the intensive scale‐up was jointly implemented by the Government of Nigeria and a consortium led by FHI 360. Nine of the LGAs were urban and five, rural.

The Nigeria HIV treatment guidelines in operation at the time stipulated an ART initiation cutoff of CD4 ≤500cells/μl. The 14 LGAs were supported to commence a pilot programme for “test and start” in July 2016. To exclude this period of incomparability in the standards of service provision between scale‐up and other LGAs from our analysis, we selected June 2014 as the start date and May 2016 as the end date. This allowed one year before and one year after the introduction of commART in the 14 LGAs.

For this study, using data from service provision, we treated each of the 14 LGAs as non‐randomly assigned into one of two LGA clusters based on the variant of commART implemented. We tagged these two variants: commART Model A (on‐site initiation model) and commART Model B (immediate referral model). The allocation to each model was informed by the commART package of services that was approved by the state health authorities for their LGAs. Ten LGAs (five urban, five rural) were assigned to Model A, and four LGAs (all urban) were assigned to Model B. The full details of the commART package for each model are described in the next section. The characteristics of these 14 LGAs are presented in Table 1. The estimated combined population of the LGAs is 5,602,243 individuals. Using figures obtained from PEPFAR programme projections for Nigeria in 2015, total combined HIV prevalence for these 14 LGAs was 4.7% with an estimated total of 164,391 people living with HIV across the LGAs. Of these, over 97,000 were estimated to be eligible for ART as per 2014 national guidelines (CD4 ≤500), but only 24,420 were on ART by the end of 2014, leaving an unmet need of close to 140,000 people living with HIV.

**Table 1:** Prevalence and coverage characteristics of the 14 intervention local government areas

CommART model	Local government area (LGA)	Total population^a^	HIV prevalence^b^	People living with HIV (PLHIV)^b^	Eligible for ART (total)^b^	PLHIV currently on treatment^b^	ART coverage (total)^b^	Unmet need^b^
Model A	Ikot Ekpene	193,201	7.2%	14,324	8403	3056	21%	11,268
	Okobo	140,472	10.2%	14,825	11,850	111	1%	14,714
	Oron	118,054	8.9%	10,779	5380	3142	29%	7637
	Uruan	159,721	7.2%	11,897	9178	340	3%	11,557
	Uyo	418,149	6.1%	26,480	11,624	3502	13%	22,978
	Calabar South	247,757	5.3%	14,714	10,734	1037	7%	13,677
	Calabar Municipal	231,929	3.4%	8920	2753	4383	49%	4537
	Eleme	257,799	4.7%	4922	3887	‐	0%	4922
	Obio/Akpor	627,858	3.6%	9170	3653	3653	40%	5487
	Port Harcourt	730,981	4.5%	13,561	5195	2303	17%	11,258
Model B	Ajeromi‐ Ifelodun	610,570	0.8%	5711	13,629	2676	13%	17,705
	Agege	908,219	2.0%	20,381	4566	3	0%	5708
	Surulere	288,494	1.2%	3761	3868	87	2%	4857
	Apapa	669,039	0.7%	4944	2882	127	3%	3634
Total/average		5,602,243	4.7%	164,389	97,602	24,420	14%	139,939

a Figures represent projection estimates derived from the 2006 national census figures 23 using annual growth rate of 3.5%.

b Figures are estimates obtained from PEPFAR programme projections for Nigeria in 2015.

Ethics approval was obtained from FHI 360 Office of International Research Ethics (OIRE) under the definitions of the Department of Health and Human Services Code of Federal Regulations (45 CFR part 46.102(d)(f)). FHI 360 OIRE Project # 990183‐3.

### 2.2 The intervention

In Table 2, we present the characteristics of each commART model as well as features of routine health facility service delivery. For both commART models, mobile teams were set up by the respective state governments with support from FHI 360. These teams delivered HIV services integrated into general health campaigns, which also included assessments for noncommunicable diseases. Each mobile team was comprised of trained community volunteers (one community mobilizer, five HIV counsellors and testers, four case managers, two adherence counsellors, and two data entry clerks) supervised by formal healthcare professionals (one doctor, one pharmacist, and one laboratory scientist/technician). Based on each LGA's HIV prevalence and population, we estimated the weekly rates of HIV testing and counselling required to identify the projected number of people living with HIV over a 12‐month period of implementation. We deployed between three and six teams per LGA based on this estimate, and these teams delivered the package of services approved for that LGA. All mobile teams were linked to designated hub health facilities through which they received health commodities and supplies for community service delivery. LGA health departments and private entities, such as pharmaceutical companies, also provided health commodities beyond HIV for the integrated general health campaigns.

**Table 2:** Comparative summary of HIV service delivery models

HIV Care Cascade	Routine facility HIV service delivery	CommART model A – on‐site initiation model	CommART model B – immediate referral model
Demand creation	Routine counselling and testing services/provider‐initiated testing & counselling (PITC)	Community mobilization, LGA enumeration and saturation/community outreaches	Community mobilization, LGA enumeration and saturation/community outreaches
Patient access	Clients self‐present to hospital to seek medical care	Mobile multidisciplinary teams conduct house‐house testing and provision of services in community	Mobile multidisciplinary teams conduct house‐house testing and provision of services in community
HIV counselling and testing	Pre‐test and post‐test counselling conducted with the test. Written informed consent obtained. Can take up to 15 minutes. In group settings, group information may apply	Pre‐ and post‐test counselling alongside HIV test. Informed consent obtained Can take up to 10 minutes. In group settings, group information may apply	Pre‐ and post‐test counselling alongside HIV test. Informed consent obtained. Can take up to 10 minutes. In group settings, group information may apply
Providers	Formal health care providers trained routinely on the job	Task‐sharing: formal providers with community lay workers. Two weeks’ basic role‐specific training on community ART model/competence assessment done prior to selection and continued after that. Constituted into mobile teams under supervision of a trained doctor/nurse	Task‐sharing: formal providers with community lay workers. Two weeks’ basic role‐specific training on community ART model/competence assessment done prior to selection and continued after that. No pharmacists on mobile team
Enrolment for HIV positives	Enrolment into national tools and registers	Enrolment into national tools and registers	Enrolment into national tools and registers
Lab and clinical evaluation	Routine via labs. Results ready at the next clinic visit	Clinical evaluation by mobile team physician, lab evaluation using point‐of‐ care (POC) equipment or sample referral. Same day results	Clinical evaluation by mobile team physician, lab evaluation using point‐of‐care (POC) equipment or sample referral. Same day results
Eligibility criteria for ART	Per national guideline (CD4<500 or WHO stages 3 and 4) for (adults and adolescents). For children – all HIV‐positive children under age five years	Per national guideline (CD4<500 or WHO stages 3 and 4) for (adults and adolescents). For children – all HIV‐positive children under age five years	Per national guideline (CD4<500 or WHO stages 3 and 4) for (adults and adolescents). For children – all HIV‐positive children under age five years
Adherence preparation	Complete three sessions at three separate visits	Complete three sessions at identification within community. Case managers assigned for immediate follow‐up starting the same day	First session at the community. Other sessions to be completed in the facility
Place and time of ART initiation	Within health facility, after three sessions of adherence. Can take up to three weeks from HIV diagnosis	At the point of identification within community. ART commenced immediately after completing three sessions of adherence following readiness preparation and determination. Can take up to three hours	Within health facility, after abridged sessions of adherence. Usually done same day
Follow‐up care	One monthly first visit, three monthly after that except otherwise stated	Phone calls/SMS/home visit every three days during first two weeks. Follow‐up visits to community team at two weeks and one month after ART initiation. The subsequent linkage is made to the static facility	One monthly first visit, three monthly after that except otherwise stated

The main differences between both models regarding HIV service provision related to place and time of ART initiation, time of linkage to the hub health facility, and the number of contacts for care and follow‐up within the first month after ART initiation. While Model A offered HIV services within communities, from HIV diagnosis to immediate ART initiation and some follow‐up, Model B offered only services for HIV diagnosis up to baseline evaluation and referral for ART initiation to nearest health facility providing HIV services. For Model A, clients were linked to the hub health facility one month after successful ART initiation and follow‐up visits within the community, while those in Model B were linked immediately after initial baseline evaluation for ART initiation and continued care at the hub health facility. Thus, for Model A, HIV services from diagnosis, ART initiation, and one‐month follow‐up occurred simultaneously within facilities and in communities with community‐based patients linked to the health facility after that for continued care. In Model B, all ART initiations and follow‐up took place at hub health facilities regardless of time and place of HIV diagnosis.

In April and May 2015, community volunteers were recruited through a community consensus process involving the local authorities and community leaders using jointly agreed eligibility criteria based on the scope of work to be performed. Recruited clinicians, nurses, laboratory staff members and pharmacists received orientation on basic HIV care, diagnosis, clinical and laboratory evaluation, adherence preparation, as well as ART initiation and follow‐up. We conducted a one‐week task‐specific training and planning meeting for the community volunteers using training modules adapted from the national guidelines, followed by a week of practical assessment in the field. Only a good performance guaranteed participation in the programme. Planning meetings involved geographical mapping of each LGA per political wards, settlements, and enumeration areas, and development of schedules for implementation. We leveraged information from maps and microplans already developed and in use by LGAs for routine community‐level immunization coverage.

### 2.3 Control LGAs

For this study, we selected LGAs that did not implement commART and allocated them into two groups to serve as control clusters for the two intervention models. The control LGAs were matched to intervention LGAs on (a) geographical location‐control LGAs were selected from within the same Nigerian states as the intervention LGAs, (b) baseline level for the two outcomes, and (c) baseline trend for the two outcomes. We defined baseline comparability as having a *p*‐value greater than 0.1 on both baseline level and baseline trend for the two study outcomes. This process generated 32 LGAs as the control for Model A cluster and two LGAs as the control for Model B cluster.

### 2.4 Data collection

We extracted aggregate data from the Nigeria District Health Information System (DHIS) platform for LGA‐based HIV services from all health facilities providing HIV services within all the study LGAs for the period June 2014 to May 2016. DHIS is a national database used to manage data from routine health service provision in health facilities. During service provision at commART locations and static health facilities, data were entered using nationally approved paper‐based data collection and reporting tools. These are transcribed, daily, by documentation clerks into national service registers domiciled at the HIV care facilities (and therefore captures LGA wide enrolments from both community and facility). CommART entries in the registers were assigned a code to differentiate community HIV care‐enrolments from facility‐based enrolments. Every month, the service registers were validated and summarized into national Monthly Summary Forms (MSFs) from where summary information was made into DHIS software as data elements and transmitted electronically into the Government of Nigeria's DHIS platform. Indicators on the identification of people living with HIV were number of pregnant women tested HIV positive and number of individuals who tested HIV positive. Indicators for people living with HIV that were initiated on ART were the number of HIV‐positive pregnant women newly initiated on ART for their own health during the reporting period and number of individuals newly initiated on ART during the reporting period.

### 2.5 Statistical analysis

The main tool for the study was interrupted time‐series analysis (ITSA) for single and multiple group comparisons, implemented in STATA v12, using ordinary least squares regression with Newey‐West standard errors. Time‐series used standardized monthly service data of LGA groups from about one year before to one year after commART introduction (June 2014 to May 2016). Monthly data for each LGA was standardized per 100,000 catchment population using each LGA's estimated population. The standardization was to ensure that inferences from the analyses are based on internally comparable data between all groups of LGAs. We then summed individual LGA figures per the four LGA clusters: Model A (10) and control (32) LGAs; Model B (4) and control (2) LGAs.

The single and multiple group analyses models are specified respectively as follows:

Y_t_ = β_0_ + β_1_T_t_ + β_2_X_t_ + β_3_X_t_T_t_ + ε_t_

Y_t_ = β_0_ +β_1_T_t_ + β_2_X_t_ + β_3_X_t_T_t_ + β_4_Z_t_ + β_5_ZT_t_ + β_6_Z_t_X_t_ + β_7_Z_t_ X_t_T_t_ + ε_t_

Here, Y_t_ is the total number of outcome variable at time t, per 100,000 catchment population. Time (in months) covered for this analysis was from June 2014 to May 2016. Β_0_ is the level of the outcome at the start of study period. β_1_ is the average monthly change in outcome over the pre‐commART level (attributable in this study to routine facility HIV delivery). β_2_ is the change in the level of outcome in the period immediately after commART introduction. X_t_ designates commART introduction period. β_3_ is the difference in the outcome trend after commART introduction, compared with the monthly trend before. β_4_ is the difference in level of outcome between intervention and control LGA groups prior to commART introduction; Z_t_ designates the period before commART introduction. β_5_ is the difference in baseline trend of outcome between intervention and control LGA groups prior to commART introduction; β_6_ indicates difference in the level of outcome between intervention and control LGAs immediately after commART introduction; β_7_ indicates difference in outcome trend between intervention and control LGAs following commART introduction, compared to pre‐commART trends. T_t_ is the time since start of the study and ε_t_ is the random error term. Linear trends following commART introduction for single group comparison is β_1_ + β_3_ for intervention LGAs. For multiple group comparison, linear trend post commART introduction is β_1_ + β_5_ + β3 + β_7_ for intervention LGAs and β_1_ + β_3_ for control LGAs; while β_5_ + β_7_ is the difference in trends between both intervention and control clusters. A Prais‐Winsten regression was run after each analysis to check the Durbin‐Watson statistic (DW stat) for autocorrelation (DW stat ≤1.5 or ≥2.5). Statistical significance was set at *p*<0.05. Secondary analyses were done to compare changes in facility‐ and community‐based outcome levels.

## 3 Results

### 3.1 Enrollment into HIV care: number of HIV‐positives identified and number of persons initiated on ART

Both Model A and Model B clusters had more HIV‐positive individuals identified and initiated more HIV‐positives on ART per 100,000 catchment population in the 12 months after commART introduction compared to the 12 months before (Model A: HIV‐positives 11,374 *vs*. 5352, ART initiations 7347 *vs*. 2181 and Model B: HIV‐positives 907 *vs*. 383, ART initiations 499 *vs*. 152) (for breakdown into community/facility component, see Figure S1).

### 3.2 Initiation on ART of persons diagnosed HIV positive

The overall total number of HIV‐positives transitioned to ART irrespective of mode of enrollment increased in both Model A and Model B following the introduction of commART: 64.08% vs. 39.85% for Model A and 54.63% vs. 40.21% for Model B (Figure S1).

For Model A cluster, 59.55% of HIV‐positives identified in health facilities after commART introduction transitioned to ART compared to 39.85% before commART introduction. On the other hand, 69.12% of HIV‐positives identified within communities following commART introduction transitioned to ART.

For Model B cluster, 80.92% of HIV‐positives identified in health facilities after commART introduction transitioned to ART compared to 40.21% before commART introduction. On the other hand, following commART introduction, only 31.61% of HIV‐positives identified within communities and referred for treatment transitioned to ART.

**Figure 1:**
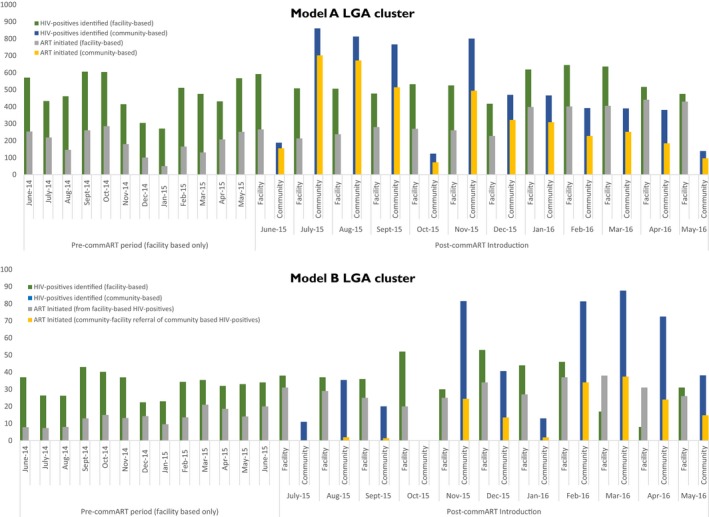
Enrolment into HIV care: number of persons identified HIV positive and number of ART initiations by month, community/facility contribution; standardized per 100,000 catchment population.

Figure 1 shows facility and community contributions for intervention LGAs over the study period.

### 3.3 Impact of commART on number of HIV‐positive persons identified and number of persons commenced on ART

#### 3.3.1 Model A cluster (single group comparison of before‐and‐after trends within Model A LGAs only)

**Figure 2:**
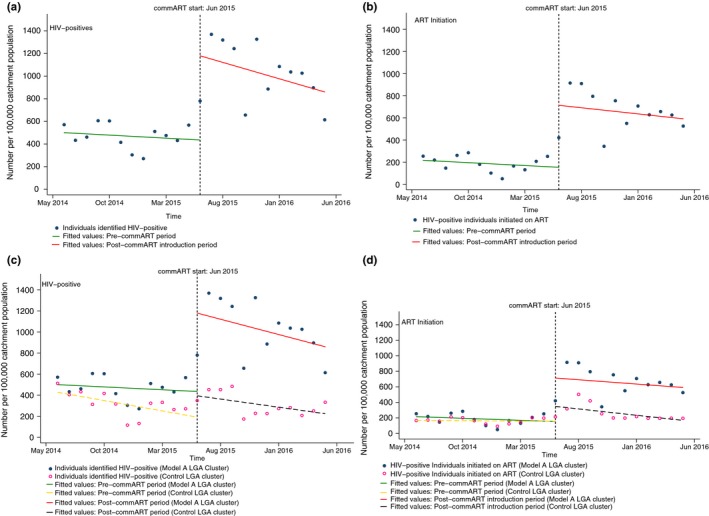
Single and interrupted time‐series analysis: commART Model A and control local government area (LGA) clusters.

Identification of HIV‐positive persons at the start of the period under review was 500 per 100,000 catchment population, with a nonsignificant decrease in about five persons per month before commART introduction. In the first month of commART introduction, we noted a significant increase in the number of people identified: 744 HIV‐positive individuals per 100,000 catchment population (*p* = 0.00, 95% CI: 360.35 to 1127.77) (Table 3). Compared to the trend before commART introduction, we noted a nonsignificant decrease in 23 HIV‐positive individuals identified per 100,000 catchment population per month (see Figure 2A). The overall trend following commART introduction for identification of HIV‐positives was not significantly different from zero.

**Table 3:** Regression parameters – Model A and Control LGA Clusters

	No. of individuals tested HIV positive				No. of HIV‐positive individuals initiated on ART			
	Coefficient	T‐stat	*p*‐value	95% CI	Coefficient	T‐stat	*p*‐value	95% CI
**CommART Model A LGA cluster**								
β_0_: intercept	500	10.35	0.00	399.66 to 601.41	216.41	7.09	0.00	152.72 to 280.10
β1: pre‐commART implementation trend	−5.42	−0.72	0.48	−21.12 to 10.29	−5.24	−0.98	0.34	−16.40 to 5.91
β2: commART effect on level immediately after introduction	744.06	4.04	0.00	360.35 to 127.77	560.10	3.90	0.00	260.56 to 859.64
β3: commART effect on trend after introduction (relative to pre‐introduction)	−23.63	−0.98	0.34	−73.74 to 26.48	−5.82	−0.34	0.74	−41.84 to 30.19
β1+β3: Linear trend post commART introduction	−29.05	−1.27	0.22	−76.63 to 18.53	−11.07	−0.67	0.51	−45.31 to 23.17
**CommART Model A LGA and Control LGA cluster**								
β4: indicator level difference before commART introduction	72.35	1.18	0.24	−51.27 to 195.97	47.51	1.42	0.17	−20.32 to 115.34
β5: indicator slope difference before commART introduction	14.30	1.51	0.14	−4.87 to 33.47	−4.20	−0.70	0.49	−16.38 to 7.98
β6: indicator level difference after commART introduction	541.07	2.73	0.01	140.60 to 941.54	367.69	2.29	0.03	42.73 to 692.64
β7: indicator slope difference after commART introduction (pre‐post trend change difference)	−27.88	−1.09	0.28	−79.77 to 24.01	9.51	0.49	0.62	−29.40 to 48.42
**Post intervention trend**								
β1 + β5 + β3 + β7: Linear trend post‐commART (Model A LGA cluster)	−29.05	−1.27	0.21	−75.15 to 17.05	−11.07	−0.67	0.50	−44.24 to 22.11
β1 + β3: Linear trend post‐ commART (Control LGA cluster)	−15.47	−2.21	0.03	−29.61 to −1.32	−16.37	−2.03	0.05	−32.65 to −0.10
β5+ β7: Difference	−13.58	−0.57	0.57	−61.80 to 34.64	5.31	0.29	0.77	−31.65 to 42.26

For persons initiated on ART (Figure 2B), 216 HIV‐positive individuals per 100,000 catchment population were being commenced on ART at the start of the period, with a nonsignificant pre‐commART decrease in five persons per 100,000 catchment population per month. Immediately after commART introduction, there was a significant increase by 560 HIV‐positives per 100,000 catchment population commenced on ART (*p* = 0.00; 95% CI: 260.56 to 859.64). Compared to the trend before commART introduction, we noted a nonsignificant decrease in five ART initiations per 100,000 catchment population per month. The overall trend following commART introduction for both outcomes was not significantly different from zero (Table 3).

#### 3.3.2 Model A and control LGA clusters (multiple group comparison)

By design, Model A and its control LGA clusters were comparable on both study outcomes for baseline level and baseline trend. Following commART introduction, we noted immediate significant differences between Model A and control LGAs in the levels of both outcomes. On average, per 100,000 catchment population, 541 more HIV‐positive persons were identified (*p* = 0.01, 95% CI: 140.60 to 941.54), and 368 more HIV‐positive individuals were commenced on ART (*p* = 0.03, 95% CI: 42.73 to 692.64) in Model A compared to control. We did not note any significant difference between both groups in the trend of both outcomes following commART introduction.

The overall trend during the post‐commART introduction period in the control LGAs represented a significant decline in the number of people identified HIV positive (−15.47, *p* = 0.03, 95% CI: −29.61 to −1.32). This was not significantly different for Model A. The trends in the post‐comm ART period for diagnosed people living with HIV commenced on ART in Model A and control LGAs were not significant within each group of LGAs nor statistically different from each other.

#### 3.3.3 Model B cluster (single group comparison)

**Figure 3:**
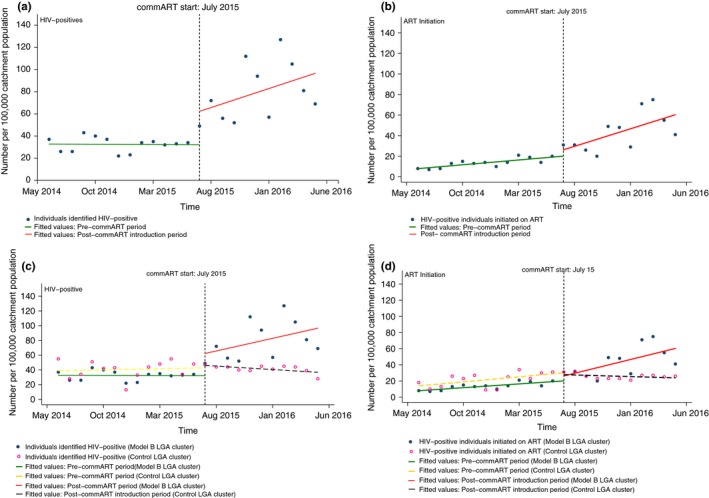
Single and multiple group interrupted time‐series analysis: commART Model B and control local government area (LGA) clusters.

In Model B LGAs, about 33 HIV‐positive persons per 100,000 catchment population were identified at the start of the study period, with a nonsignificant gradual reduction by less than one (0.05) HIV‐positive person per month prior to commART introduction. In the first month of commART introduction, we noted a significant increase in number of individuals identified by 30 HIV‐positives per 100,000 population (*p* = 0.01, 95% CI: 8.38 to 51.93). Compared to the trend before commART introduction, we noted a nonsignificant decrease in three HIV‐positive individuals identified per 100,000 catchment population per month (*p* = 0.10; 95% CI: −0.77 to 7.74). The detailed parameters are shown in Table 4, and the plots depicted in Figure 3A.

**Table 4:** Regression parameters – Model B and Control LGA Clusters

	No. of individuals tested HIV positive				No. of HIV‐positive individuals initiated on ART			
	Coefficient	t‐stat	*p*‐value	95% CI	Coefficient	t‐stat	*p*‐value	95% CI
**CommART Model B LGA cluster**								
β_0_: intercept	32.75	8.81	0.00	25.00 to 40.51	7.93	6.91	0.00	5.54 to 10.33
β_1_: pre‐commART implementation trend	−0.05	−0.13	0.89	−0.84 to 0.74	0.93	5.33	0.00	0.57 to 1.29
β_2_: commART effect on level immediately after introduction	30.15	2.89	0.01	8.38 to 51.93	6.15	1.07	0.29	−5.85 to 18.15
β_3_: commART effect on trend after introduction (relative to pre‐introduction)	3.49	1.71	0.10	−0.77 to 7.74	2.48	1.72	0.10	−0.52 to 5.47
β_1_ + β_3_: Linear trend post commART introduction	3.43	1.71	0.10	−0.74 to 7.61	3.4	2.39	0.02	0.44 to 6.38
**CommART Model B LGA and control LGA cluster**								
β_4_: indicator level difference before commART introduction	−5.97	−0.77	0.45	−21.65 to 9.70	−5.93	−1.86	0.07	−12.39 to 0.52
β_5_: indicator slope difference before commART introduction	−0.36	−0.39	0.70	−2.26 to 1.52	−0.31	−0.87	0.39	−1.06 to 0.42
β_6_: indicator level difference after commART introduction	26.83	2.19	0.03	2.03 to 51.62	8.48	1.22	0.23	−5.57 to 22.54
β_7_: indicator slope difference after commART introduction (pre‐post trend change difference)	4.74	2.09	0.04	0.16 to 9.31	4.13	2.72	0.01	1.06 to 7.19
**Post intervention trend**								
β_1_ + β_5_ + β_3_ + β_7_: Linear trend post‐commART (Model A LGA cluster)	3.44	1.72	0.09	−0.61 to 7.48	3.41	2.39	0.02	0.53 to 6.29
β_1_ + β_3_: Linear trend post‐commART (Control LGA cluster)	−0.94	−1.91	0.06	−1.92 to 0.05	−0.40	−1.15	0.26	−1.10 to 0.30
β_5_ + β_7_: Difference	4.37	2.12	0.04	0.21 to 8.54	3.81	2.59	0.01	0.85 to 6.77

Regarding total number of HIV‐positive persons initiated on ART, eight persons per 100,000 catchment population were being commenced on ART before commART introduction, with a significant pre‐commART trend increase in one person per 100,000 population per month (*p* = 0.00; 95% CI: 0.57 to 1.29). There was no significant immediate increase after commART introduction. Compared to the trend before commART introduction, the trend for HIV‐positives initiated on ART was not significantly different from zero. The overall trend during the post‐commART introduction period represented a significant increase in HIV‐positives initiated on ART (3.4, 95%, *p* = 0.02, 95% CI: 0.44 to 6.38). Figure 3B depicts these findings. The trend for HIV‐positives identified in this period was not significantly different from zero (Table 4).

#### 3.3.4 Model B and control LGA clusters (multiple group comparison)

By design, Model B and control LGAs had comparable baseline level and trends for both study outcomes. Following commART introduction, we noted immediate significant differences between both clusters on levels of only one outcome. Per 100,000 population, 26 more HIV‐ positive persons were identified (*p* = 0.03, 95% CI: 2.06 to 51.62). The difference on number of HIV‐positives commenced on ART was not significantly different from zero.

Model B LGAs also had a significantly higher monthly trend than the control cluster by four more HIV‐positives per 100,000 catchment population per month (*p* = 0.04, 95% CI: 0.16 to 9.31), and two more ART initiations per 100,000 catchment population per month (*p* = 0.01, 95% CI: 1.06 to 7.19) following commART introduction.

The linear trends in the post‐commART introduction period for HIV‐positives identified in either cluster was not significant but were statistically different from each other, with Model B cluster higher by 4.37 HIV‐positives (*p* = 0.04, 95% CI: 0.21 to 8.54). In the post‐commART introduction period, there was a positive linear trend in Model B LGAs for number of HIV‐positives commenced on ART (3.41, *p* = 0.02, 95% CI: 0.53 to 6.29), and this was significantly higher (3.81, *p* = 0.01, 95% CI: 0.85 to 6.77) than the linear trend for its control LGAs during the same period. Figure 3C, D, and Table 4 show these findings.

## 4 Discussion

Our study found increased total uptake for HIV services (HIV‐positives identified and ART commencements) because of commART implementation, suggesting that HIV treatment coverage can be substantially increased—even using existing treatment guideline eligibility criteria—if services are taken to communities with community involvement.

The introduction of commART Model A in the LGAs brought about significant increases in the number of people living with HIV identified and commenced on treatment compared to the pre‐commART period and control LGAs. Several model characteristics may have contributed to the degree of intervention effects. For example, the multidisease approach employed during campaigns such as blood pressure checks, drug dispensation for malaria, and free over‐the‐counter drugs, as well as collaborative community participation may have contributed to community acceptability of testing outside of health facilities and reduced the stigma barrier associated with uptake of HIV services. Our experience is consistent with other studies 7, 24 showing that such strategies are effective in overcoming stigma, cultural and other community‐level barriers across the HIV treatment cascade.

Our study found increased uptake of antiretroviral treatment among HIV‐positives identified in Model A cluster following the introduction of commART. Though facility‐enrolment accounted largely for the total cluster HIV‐positives identified, we found a higher ART uptake, both in absolute numbers and proportion, among HIV‐positives identified through community enrolment and immediate initiation. Certain elements of model design may be responsible for this differential community/facility treatment uptake. The first possible explanation may be the time and place of ART initiation. It is possible that the availability of ART for immediate initiation within the community after HIV diagnosis resulted in a higher uptake of antiretroviral treatment compared to conventional facility‐based treatment initiation, which routinely takes between two and three hospital visits after initial contact. Indeed, we found that while the facility‐based ART transition rate pre‐ and post‐commART introduction increased from 39.85% to 59.55%, the community‐based ART transition was 69.12%. Our finding is consistent with findings of a randomized controlled trial 25 by MacPherson and colleagues among 16,600 Malawian adults that showed a significant increase in ART initiation among those offered optional home ART initiation following home testing. The apparent advantage of this model of commART appears to be conferred by its design allowing easy integration of all demand‐and‐supply processes at the point of care delivery. This comprehensive design feature helps overcome access barriers that may exist, especially financial and geographic. These results strongly suggest that inadequacies exist in the “facility‐alone” model of delivery for ART scale‐up. This finding also has implications for impending “test and treat” guidelines. While immediate ART initiation, or as soon as possible following HIV diagnosis, will be expected to get antiretroviral treatment to more people who need it, this strategy will need to be combined with innovative approaches and channels that deliver care and treatment beyond the boundaries of healthcare facilities.

In Model B cluster where all ART initiations occurred in the health facility, we noted that increased cluster uptake of antiretroviral treatment among HIV‐positives following commART introduction was largely accounted for by HIV‐positives diagnosed in health facilities. This is shown by an increase in the month‐on‐month ART initiations among HIV‐positives enrolled through health facilities, despite higher increases in HIV‐positives diagnosed from the communities. This indicates that while Model B may have been effective at increasing identification of people living with HIV within communities, there was significant attrition between HIV‐positives identified and referred from the communities and those that eventually completed referrals at the facility for ART initiation. This effect would be unexpected given that all the Model B LGAs are urban with better physical access to hub health facilities. On the other hand, it could indicate that, despite HIV services being provided free, the added financial cost of self‐transportation to the hospital may constitute some barrier to ART commencement. More important, this would represent an inherent weakness in the design of this model that programme managers should be aware of *ab initio*. Elements of programme design to mitigate this potential leakage include escorted referrals and making transportation available from community testing points to hub health facilities. Holmes and colleagues also described other approaches to improve community‐facility linkages for improved HIV cascade progression 26. If well implemented, studies 7 have shown that such facilitated linkages to treatment have equal or higher ART initiation rates compared to facility‐based ART initiation.

The observation of commART accentuation of month‐on‐month increase in ART initiation in Model B deserves further comments. While, as discussed previously, this effect was not directly due to commART, the intervention may have indirectly mediated this outcome nevertheless. Providing, on a massive scale, community‐level information, education, and communication, as well as stigma‐reduction messages, may have resulted in many undiagnosed people living with HIV who choose to independently seek out HIV testing services at health facilities, who may then subsequently experience delays between HIV diagnosis and ART commencement. This delay mechanism may have given rise to the observed pattern of significant trend increase in ART initiation despite the lack of trend increases in HIV‐positives identified. This delay is not surprising given well‐documented time delays in time to ART initiation following HIV diagnosis and care enrolment in sub‐Saharan Africa 27. This further highlights the apparent weakness in this model and makes a case for ensuring stronger facilitated linkages when deploying such single HIV testing interventions. More important, this may also indicate that facility‐based delivery still has higher absorptive capacities waiting to be utilized. Studies have shown that strategies to remove facility‐level barriers (such as reduction in long waiting times) in antiretroviral treatment programmes often result in increased uptake of treatment 28. While scaling up ART access through community approaches is important, the opportunities for improving facility ART uptake should not be overlooked.

The relative consistency of both community models in increasing access and uptake of HIV testing and ART services provides some assurance that large‐scale community interventions do hold some promise in contributing to meeting the global access target of eliminating HIV by 2030. The design of integrating HIV services into the general campaigns may have contributed to this success as it bypasses the stigma associated with pure HIV services. We opine that this should be a key feature of all community‐based HIV services, especially where stigma may be an issue. On the other hand, the future impact that such an entry route into HIV care may have on retention in care and losses to follow‐up, upon which arguments 27 against community involvement are based, indicates that a lot more needs to be known about how communities can optimally improve HIV service delivery. What is known for now is that some inadequacy exists in the facility‐alone model of care to achieve epidemic control by universal ART access.

This study has limitations. For cascade progression, our model did not consider the number of ART commencements as a proportion of HIV‐infected individuals that were eligible for ART using the national guidelines. We instead treated ART commencements as a proportion of all positive persons identified regardless of ART eligibility in the national guidelines. This has the effect of giving an impression of a low level of ART commencements. We, however, adopted this approach as it represents the ideal proposed in the 90‐90‐90 targets and imminent test‐and‐treat guidelines. Also, we treated all clusters uniformly in our analysis. Second, we did not randomly allocate the intervention LGA groups to models. This may have introduced some risk of bias. We attempted to minimize this risk by cluster‐matching with control LGAs with baseline similarity from within the same states and using interrupted time‐series analysis (ITSA). Third, we used routine service delivery data. Even though we standardized data achievements using prevalence and population estimates, we can only rely on available data as is. Fourth, we used aggregated data for the interrupted time‐series analysis with LGA clusters as the unit of analysis, and therefore we did not explore heterogeneity among individual LGAs. We also assumed that there were no concurrent interventions with commART that could have influenced the outcomes we evaluated. Finally, our study did not examine costs nor did we compare the efficiency or yield of HIV testing in the facility and community.

## 5 Conclusions

Our study has shown community ART models are important in optimizing the population‐level effectiveness of HIV treatment scale‐up. On‐site provision of the full range of HIV services integrated within general health campaigns in communities had a stronger effect in increasing identification of people living with HIV and their linkage to ART. Facility‐based ART delivery, while important, is insufficient to provide the service coverage necessary to achieve epidemic control. This is due to the myriad of barriers to regular healthcare access. An optimal combination of community and facility‐based approaches adapted to local epidemic conditions is, therefore, necessary to achieve the identification and linkage to HIV prevention, care, and treatment in Nigeria and possibly sub‐Saharan Africa.

## Competing interests

No competing interest declared.

## Authors’ contributions

EAO, CO, HK and KT conceived the study design and research question. OAB, MA and EAO developed the protocol. EAO, CO, DO, MA, HK, ORNM, MA, GO and OIO oversaw study implementation and data collation. OAB conducted the data analysis, literature search and wrote the first draft of the manuscript. EAO, OAB, MA, DO, EO, EJ and NO provided critical reviews of all draft versions of the manuscript. EO, EJ, HK, KT, OIO and SRP provided critical reviews of all drafts. EAO, KT, HK and SRP assisted with conducting data analysis, data interpretation and critical review of the manuscript. All authors read and approved the final manuscript.

## Supporting information


**Figure S1.** Breakdown of monthly local government area cluster achievement by facility and community contribution.
